# Early learning curve changes in objective performance indicators during robotic cholecystectomy

**DOI:** 10.3389/fsurg.2025.1679666

**Published:** 2025-10-10

**Authors:** Derrick Liu, Mallory Shields, Catherine Stricklin, Casey Troxler, Anthony Jarc, Richard Feinn, Leland Soto

**Affiliations:** 1 Frank H Netter MD School of Medicine, Quinnipiac University, North Haven, CT, United States; 2 Intuitive Surgical Inc, Sunnyvale, CA, United States; 3 Department of General Surgery, Griffin Hospital, Derby, CT, United States

**Keywords:** objective performance indicators, robotic cholecystectomy, learning curve, transition from laparoscopic to robotic cholecystectomy, ligation/division of the cystic duct

## Abstract

Learning curves for experienced laparoscopic surgeons transitioning to the robotic platform are still unknown. With the new availability of objective performance indicators (OPIs), which provide information on surgical behavior, we identified when a surgeon becomes proficient in transitioning from laparoscopic to robotic technique. As more operations were performed, the time all four robotic arms moved decreased for cystic duct ligation/division (*p* = 0.042), master clutch use increased for cystic artery ligation/division (*p* = 0.009), and camera velocity, acceleration, and smoothness increased for multiple steps. CUSUM analysis generated a learning curve for idle time, with proficiency attained after 22 operations. As the first study to evaluate objective metrics throughout a learning curve for newly performing robotic cholecystectomy, we identify relevant OPIs that may be critical for future proficiency tracking, 8 of which impact a surgical step with a steep learning curve in transitioning from laparoscopic to robotic cholecystectomy, cystic duct ligation/division.

## Introduction

The use of robotics in common general surgical procedures, such as cholecystectomy, has grown in the last ten years (1). However, there is little data about how specific surgical behaviors during specific steps of cholecystectomy change during the surgeon's transition from laparoscopic to robotic surgery, especially for surgeons who are experienced in open or laparoscopic techniques. This study provides these data through examining changes in objective performance indicators (OPIs) and, importantly, further identifies OPIs that are appropriate proxies for tracking when surgeons have attained proficiency during this transition. This type of analysis allows for more goal-directed training of surgeons.

The ability to evaluate a surgeon's technical skills without bias has been an educational goal for decades. The importance of understanding a surgeon's operative skill was illustrated by Birkmeyer et al. In this study, video assessment graded operating skills for laparoscopic bariatric surgery. Greater skill was shown to be associated with fewer postoperative complications and lower rates of reoperation, readmission, and visits to the Emergency Department (2). While this approach can be used to assess a surgeon's overall operating skills, video assessment is incredibly tedious and can introduce bias due to the skewed attention of the evaluators. Other tools that have been used to evaluate intraoperative laparoscopic and robotic skills are the Global Operative Assessment of Laparoscopic Skills (GOALS) and the Global Evaluative Assessment of Robotic Skills (GEARS), respectively (3–6). Despite these efforts to standardize the evaluation of operative proficiency, subjectivity remains.

One unbiased type of assessment during robotic surgery is metrics called OPIs. OPIs are relatively new, so studies involving them have only recently been developed. OPIs can be calculated directly from robotic data streams. They have been used to assess surgical skill and give objective, automated feedback in multiple settings. On a clinical level, they have been used to predict postoperative patient outcomes and measure intraoperative efficiency (7). OPIs have clearly been demonstrated to differ between expert and novice surgeons, indicating that OPIs change across a surgeon's learning curve. For cholecystectomy specifically, studies that assess OPIs are emerging, but very few OPI studies have identified learning curves. No studies have investigated the transition from a laparoscopic approach to mastery on a robotic platform (8).

Some work has been done to construct learning curves for laparoscopic cholecystectomies using the cumulative sum (CUSUM) analysis (9, 10). The CUSUM analysis is a statistical technique that is used to monitor changes in a process over time by continuously adding up the deviations from a target value. Consequently, CUSUM analyses can detect small changes because they track the sequential cumulative performance of any binary or continuous variable. The operation number for the peak of the CUSUM curve represents the number of operations that need to be performed to obtain proficiency. Case duration is usually the metric used in CUSUM due to its wide availability. However, there are now alternative metrics collected as OPIs that may be more accurate and could be used to identify earlier or later learning curves. This information may help determine when a surgeon becomes proficient in robotic techniques. We examined the total duration of each operation along with both step duration, active time, and idle time for each of the seven steps of robotic cholecystectomy.

Although the learning curve for laparoscopic cholecystectomy has been evaluated in several studies, the learning curve for an expert in laparoscopic cholecystectomy transitioning to robotic cholecystectomy has yet to be defined (11). Describing the learning curve for robotic cholecystectomy using OPIs may assist in the identification of early changes that are occurring as technical skill is acquired. We herein use OPIs, which are novel metrics, to assess learning changes and describe the learning curve in robotic cholecystectomy for a surgeon experienced in laparoscopic cholecystectomy who transitions to the robotic platform.

## Methods

This study includes 33 robotic cholecystectomies performed by a single surgeon who is experienced in laparoscopic cholecystectomy (>1,000 cases). These operations were the 7–39th robotic cholecystectomies that the surgeon performed from October 2020 to January 2022. Each operation utilized four robotic arms using 8 mm ports, placed diagonally across the abdomen from the right lower quadrant to the left upper quadrant. From patient right to left, the instruments that were used were Cadiere (arm 1), Camera (arm 2), Permanent cautery hook (arm 3), and ProGrasp (arm 4). Instrument exchanges occurred for round tip scissors and medium-large clip appliers through port 3. Clip ligation and division of the cystic duct and artery occurred after obtaining the critical view. Specimens were placed into a bag, and extraction occurred through port 3. Professional annotators from Intuitive Surgical, who did not perform any data analysis in this study to avoid introducing bias, collected the data on objective performance indicators (OPIs) and split each cholecystectomy into seven steps: initial exposure, removal of adhesions (if necessary), dissection of Calot's triangle, ligation/division of the cystic duct, ligation/division of the cystic artery, dissection of the gallbladder off the liver bed, and specimen removal. For each of these steps, data on hundreds of OPIs were collected by the Intuitive Data Recorder. The collected event, kinematic, and temporal OPI data are displayed in Table 1.

**Table 1:** Event, kinematic, and temporal OPI data collected.

Type of OPI	OPI name	Description	Units
Event	Number of Master Clutch Presses	Number of times the finger and pedal clutch buttons were pressed to readjust the surgeon's arm positions while the instrument arms stay immobile in the patient's body	N/A
Event	Number of Times Camera Moved	Number of times the position of the camera was adjusted	N/A
Event	Number of Arm Swaps	Number of times a surgical instrument on any robotic arm was swapped out for another	N/A
Event	Number of Instrument Energy Activations	Number of times the energy pedals were pressed for cutting	N/A
Kinematic	Path Length	Total linear distance traveled by a robotic arm	Meters
Kinematic	Total Angular Distance	Total distance traveled by different joints of the wrist (roll, pitch, yaw)	Radians
Kinematic	Angular Speed	Angular speed of the roll, pitch, and yaw joints of the wrist	Radians per second
Kinematic	Linear Speed	Speed at which a robotic arm moved	Meters per second
Kinematic	Acceleration	Rate at which the linear speed of a robotic arm changed per second	Meters per second^2^
Kinematic	Smoothness	Rate at which acceleration of a robotic arm changed per second	Meters per second^3^
Kinematic	Distribution of Linear Speeds	The range of linear speeds for the instrument tip on a given robotic arm, represented by various percentiles (1st, 25th, 50th, 75th, and 99th)	Meters per second
Temporal	Step Duration	Total time taken to complete the step of the procedure	Seconds
Temporal	Total Active Time	Total time that any of the four robotic arms was moving	Seconds
Temporal	Total Active Time of Wrist	Total time that the wrist of a given robotic arm was moving	Seconds
Temporal	Idle Time	Total time when all four robotic arms were not moving while surgical instruments were being swapped	Seconds

This data was collected across all 4 instrument arms, left and right hands at the surgical console, and from the system itself. Angular movements are defined as follows. Roll: axial rotation of the instrument wrist, Pitch: up and down rotation of the instrument wrist, Yaw: left and right rotation of the instrument wrist.

Demographic data such as age, body mass index (BMI), ASA physical status, length of surgery, time of anesthesia recovery, length of stay (LOS) in hospital, and number of post-op ED visits were collected for each patient. These data were split into tertiles to allow for examination of variances in patient characteristics as surgeon experience increased.

Subgroup analysis of all data was performed to compare differences between the indications for surgery. Each cholecystectomy was assigned to either an acute (acute cholecystitis, acute biliary pancreatitis, and choledocholithiasis) or non-acute indication (calculus of the gallbladder without acute cholecystitis). There were 11 acute and 22 non-acute cases in this study. ANOVA tests were run to compare length of surgery and length of hospital stay across the three tertiles for all cases, amongst acute cases, and amongst nonacute cases separately. This quantitative data is displayed in Supplementary Table 1.

The following statistical analyses were performed by authors with an affiliation with the Frank H. Netter School of Medicine. The values for hundreds of OPIs compared to operation number in the 33 robotic cholecystectomies were first analyzed using a Spearman rank correlation, with an alpha value of 0.05, in RStudio. Correlation coefficients along with their *p*-values were recorded. Additionally, various OPIs (total duration of each operation, along with both total duration and idle time for each of the seven steps) were analyzed using a CUSUM analysis to determine whether proficiency can be established after performing a distinct number of cholecystectomies. Spearman rank analysis was also performed for acute vs. non-acute cases in RStudio.

Because the data set was very large, to visualize macroscopic changes in event, temporal, and kinematic OPIs during specific steps, the correlation coefficients and corresponding *p*-values from the Spearman rank analysis of OPIs were represented in correlation plots created in RStudio. OPIs and the seven cholecystectomy steps are listed as row and column headings, respectively. The plots show the strength of correlation and the presence/absence of statistical significance for each OPI during each of the steps.

## Results

Throughout the study, there were no conversions to open surgery. There were two patients who returned to the ER. The first patient's indication for cholecystectomy was an obstructing gallstone without cholecystitis, and she developed abdominal pain eight days after her cholecystectomy. No surgical complication was discovered, and her pain resolved without further incident. A second patient was admitted to another hospital two weeks after surgery with choledocholithiasis requiring ERCP. Additionally, all surgeries were outpatient surgeries, and the length of stay in the hospital ranged from 1.3 to 6.55 h. Across the three tertiles, there was no difference in the average length of stay that patients had in the hospital amongst both acute and nonacute cases together (*p* = 0.104), amongst just acute cases (*p* = 0.244), and amongst just nonacute cases (*p* = 0.213). Also, the length of surgery ranged from 35 to 145 min, and across the three tertiles, there was no difference in the average length of surgery, amongst both acute and nonacute cases together (*p* = 0.277), amongst just acute cases (*p* = 0.316), and amongst just nonacute cases (*p* = 0.955). Likewise, there were no differences between average age, BMI, time of recovery, ASA physical status, and number of post-op ED visits across the three tertiles when acute and nonacute cases were analyzed together and separately. The only significant finding in the demographic data that we collected for our patients was that the average BMI for acute cases, which were fairly evenly distributed through the three tertiles, was much higher for the third tertile than for the other two tertiles (Supplementary Table 1). The lack of other significant findings within the demographic data can most likely be attributed to the small sample size in our study.

**Figure 1:**
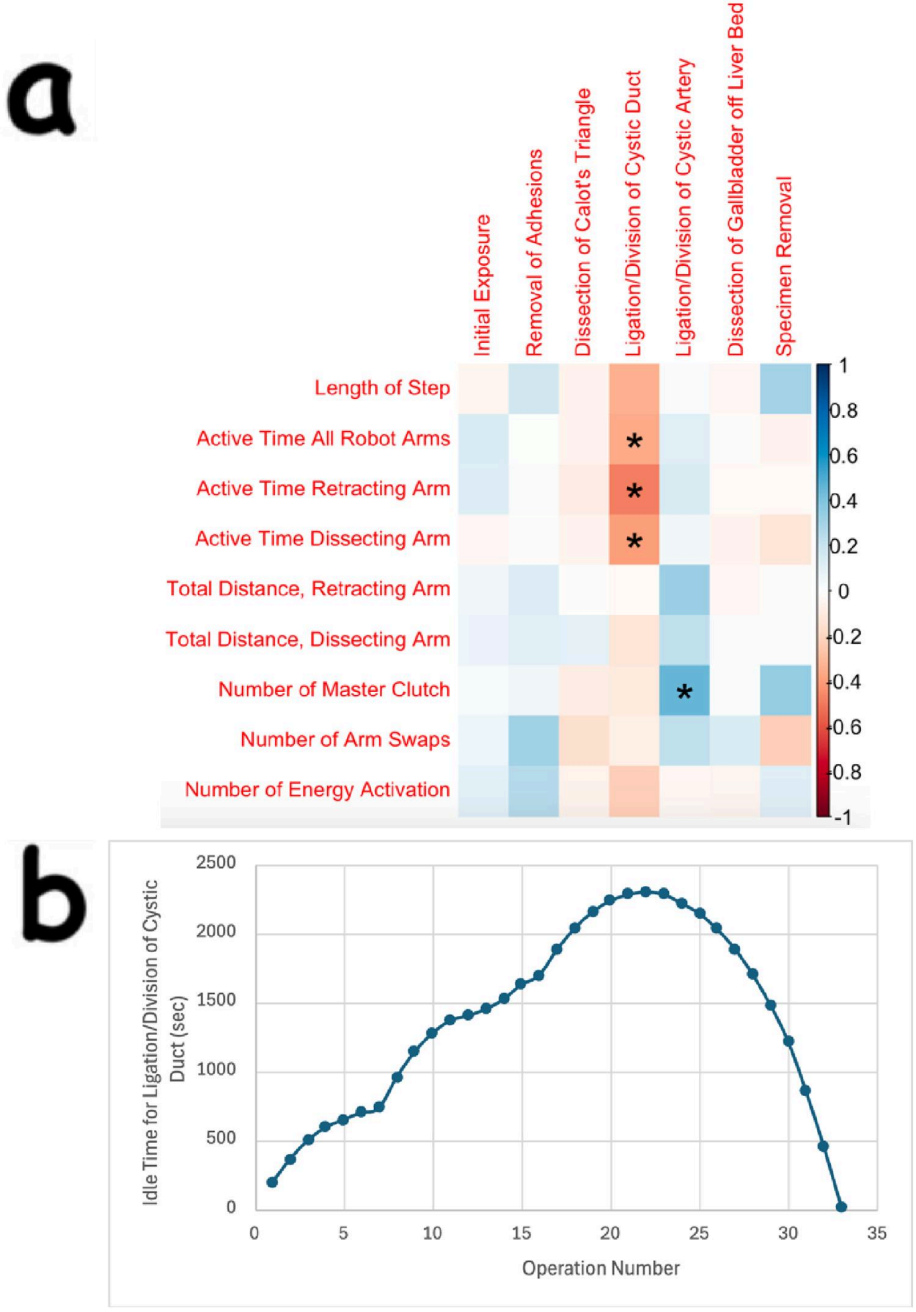
**Statistically significant findings in event and temporal OPIs for the ligation/division of cystic duct and cystic artery steps, and CUSUM learning curve for idle time in the ligation/division of cystic duct step**. **(a)** Is a correlation plot that shows statistically significant findings for various OPIs for two steps in robotic cholecystectomy. In this plot, the Event, Temporal, or Kinematic OPIs are listed as row headings, whereas the steps of robotic cholecystectomy are listed as column headings. A “*” in a box indicates statistical significance. The key for strength of correlation is displayed towards the far right, with blue indicating positive correlations and red indicating negative correlations. In **(b)**, the peak of the CUSUM learning curve for idle time in the ligation/division of the cystic duct step (at operation 22) represents the point at which proficiency is reached.

There were statistically significant changes in many event, temporal, and kinematic OPIs across multiple surgical steps. Much of the statistical significance for event and temporal OPIs was identified in the ligation/division of the cystic duct step, which requires a steep learning curve in the transition from laparoscopic to robotic cholecystectomy. During that step, as more operations were performed, the total active time of all robotic arms decreased (*p* = 0.042), the total active time of the retracting and dissecting robotic arms decreased (*p* = 0.005 and *p* = 0.023, respectively), and the reduction in step duration approached significance (*p* = 0.057). Additionally, in the ligation/division of the cystic artery step, as more operations were performed, the master clutch was used more times (*p* = 0.009). These findings are summarized together in Figure 1a.

The CUSUM analysis revealed a standard learning curve for idle time during ligation/division of the cystic duct (Figure 1b). In this step, there are 4 instrument exchanges, 3 of which are for placing clips and one for placing scissors to cut the cystic duct after ligation. This may indicate greater proficiency in clip loading, instrument exchange, and surgeon retaking control once the 22nd cholecystectomy within this study had been performed. The CUSUM analysis was also performed on the total duration of each operation, the total duration of each of the seven steps separately, and the idle time for the other 6 steps separately, but no learning curve was generated for any of these OPIs.

**Figure 2:**
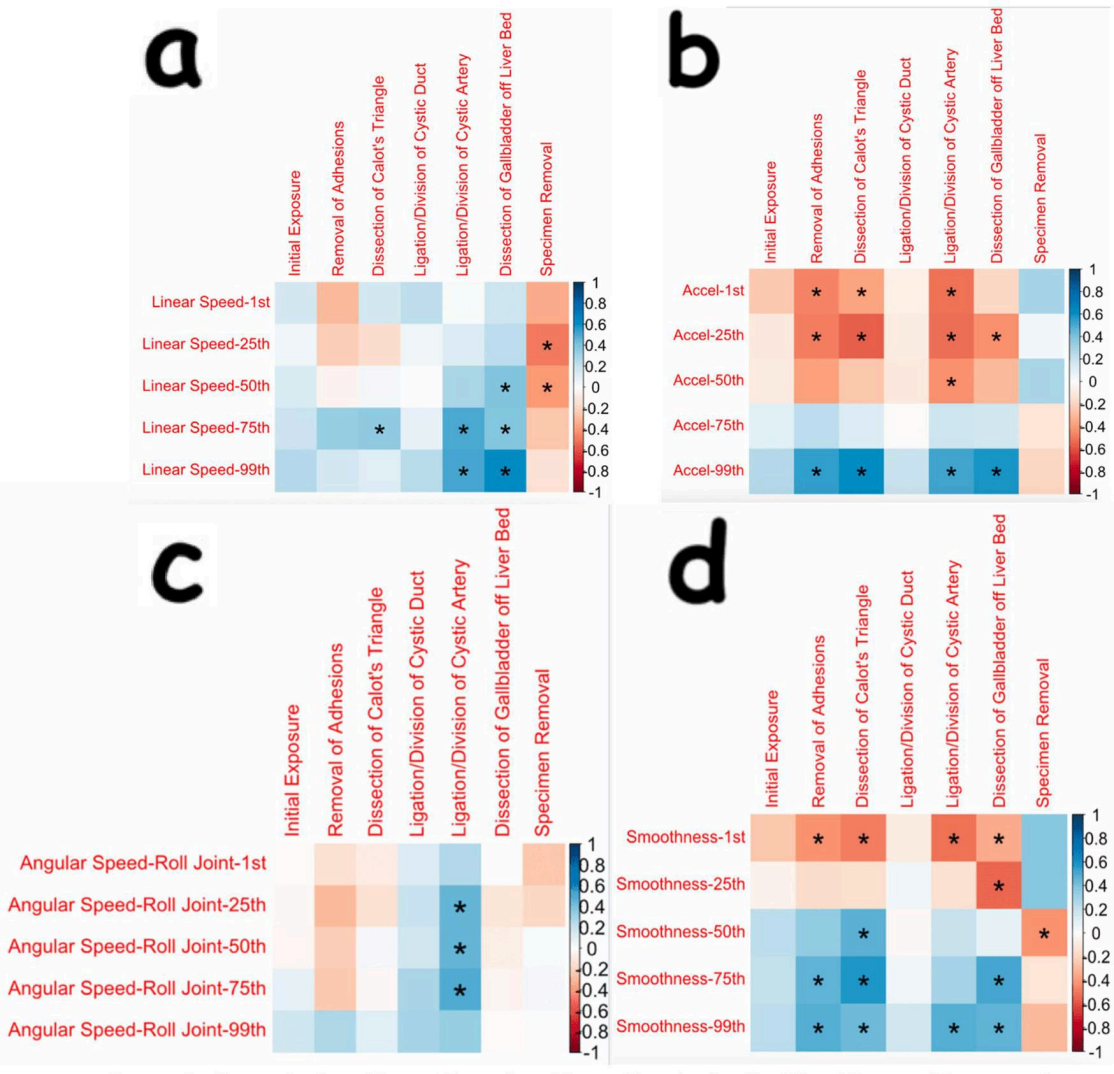
**Correlation plots showing many statistically significant changes in kinematic OPIs during distinct steps of cholecystectomy seen for the robotic arm controlling the camera**. **(a)** through **(d)** are correlation plots showing statistical significance for various OPIs, including linear speed, acceleration, angular speed, and smoothness, respectively, for the robotic arm controlling the camera. Data for the angular speed of the camera arm was recorded in the spots for the “roll joint”. The Kinematic OPIs are listed as row headings, whereas the steps of cholecystectomy are listed as column headings. A “*” in a box indicates statistical significance. “1st” represents the 1st percentile for a given measure (same for “25th”, “50th”, “75th”, and “99th”). The key for strength of correlation is displayed towards the far right, with blue indicating positive correlations and red indicating negative correlations.

There was also statistical significance in various kinematic OPIs. For example, in the removal of adhesions, dissection of Calot's triangle, ligation/division of cystic artery, and dissection of gallbladder off the liver bed steps, there were statistically significant changes in the acceleration and smoothness of the robotic arm that was controlling the camera. These findings, along with others for the camera arm, are displayed in Figure 2.

**Figure 3:**
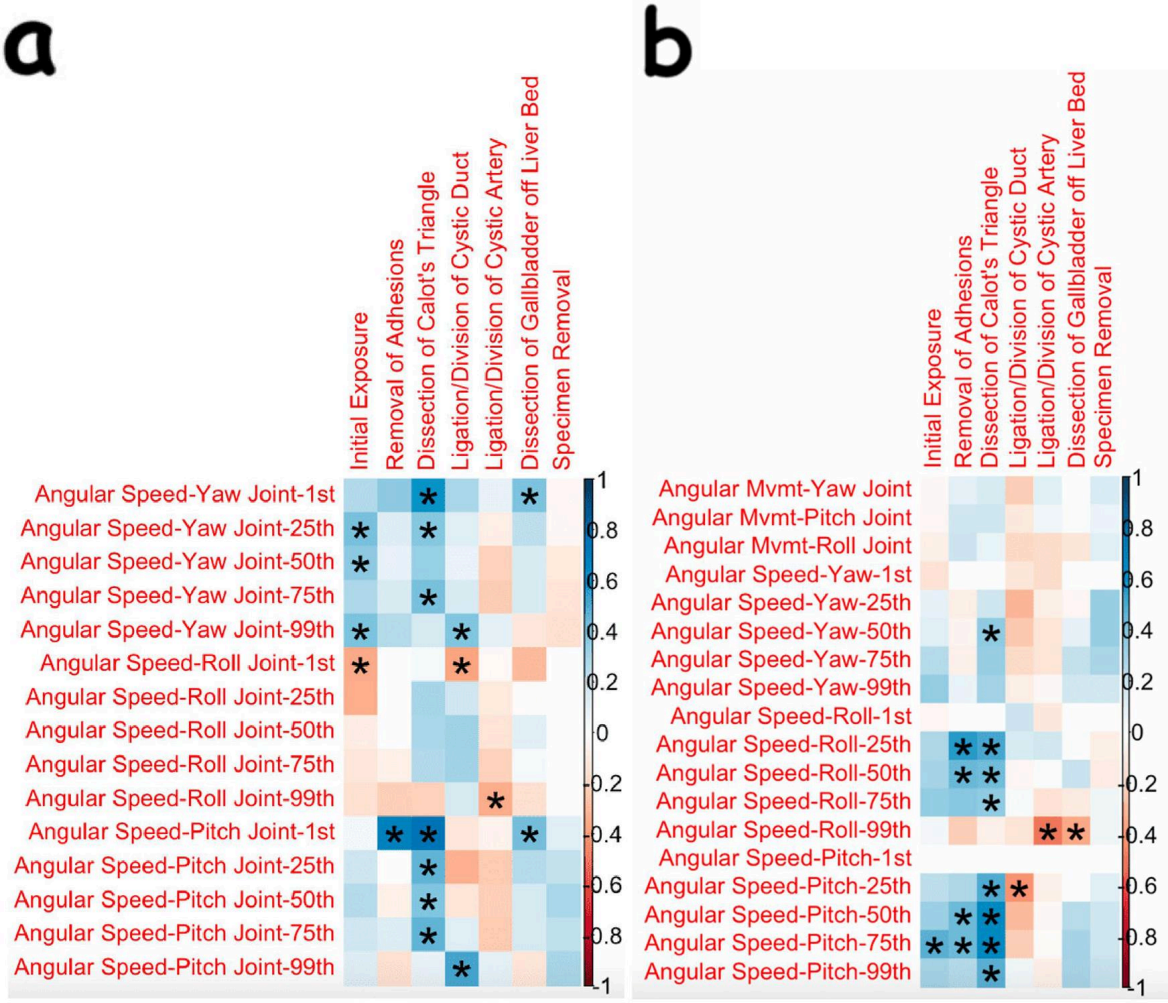
**Correlation plots showing correlations and statistical significance for angular speeds of the dissecting arm and its corresponding hand controller [(a) and (b), respectively]**. The Kinematic OPIs are listed as row headings, whereas the steps of cholecystectomy are listed as column headings. A “*” in a box indicates statistical significance. “1st” represents the 1st percentile for a given measure (same for “25th”, “50th”, “75th”, and “99th”). The key for strength of correlation is displayed towards the far right, with blue indicating positive correlations and red indicating negative correlations.

Many more angular speed OPIs had statistically significant changes for the dissecting robotic arm (and the corresponding hand controller) as compared to the retracting arm (and its corresponding hand controller). Many of the significant findings seen in OPIs during the dissection of Calot's triangle were increasing angular changes in the dissecting arm. These findings are displayed in Figure 3.

There were significant findings for the linear speed and acceleration of the retracting and dissecting arms. Of note, the acceleration of the dissecting arm increased as more operations were performed in the ligation/division of the cystic duct step. These findings are shown in Supplementary Figure 1.

There were many other kinematic OPIs that were analyzed, but statistical significance was not found (Supplementary Figure 2).

## Discussion

In this study, we aimed to investigate changes in event, temporal, and kinematic objective performance indicators (OPIs) to ultimately construct a learning curve for a surgeon adept at laparoscopic cholecystectomy who transitions to robotic cholecystectomy. We found that the most notable changes in event and temporal OPIs were seen in the ligation/division of the cystic duct step, with a decrease in the time that the instrument arms were in motion as more operations were performed. A significant increase in the use of the master clutch was seen in the ligation/division of the cystic artery step. We also found notable changes in kinematic OPIs associated with camera motion in the dissection of Calot's triangle, ligation/division of the cystic artery, and dissection of the gallbladder off the liver bed steps. Many notable changes in angular kinematic OPIs for the dissecting arm during the dissection of Calot's triangle were seen. Lastly, using CUSUM analysis, we generated a learning curve for idle time in the ligation/division of the cystic duct step, with proficiency reached at the 22nd operation.

Much of the statistical significance found for event and temporal OPIs was for the ligation/division of cystic duct, which occurs after the “critical view” is established (i.e., after the hepatocystic triangle is cleared of fat and fibrous tissue, the lower one third of the gallbladder is dissected to expose the cystic plate and only two structures are seen entering the gallbladder) (12). In this step, there is clipping and cutting, but it is unique to a surgeon experienced in laparoscopic cholecystectomy because it challenges the surgeon to have expert control of the instruments by assuming control of the instrument four times (three instrument exchanges to place clips on the duct, one for a scissor to ligate the duct). It also requires that the surgeon assume control of the clips without inadvertently activating the clip. Furthermore, new, learned motions of the hand are needed to finger clutch without squeezing the hand controls and inadvertently engaging the clip. Lastly, efficiency in this step requires the expertise of a bedside assistant who is proficient in removing and replacing instruments, and requires a surgical technologist to be facile in placing the clips on the applier.

One proxy for tracking learning in robotic cholecystectomy could be the total time the robotic arms moved. In this study, the total time that all four robotic arms moved during the ligation/division of the cystic duct decreased in a statistically significant manner as more operations were performed (Figure 1a). This finding was found in conjunction with a decreased time to complete that step that approached statistical significance as more operations were performed. Therefore, spending less time moving the robotic arms could represent the surgeon becoming more efficient with his movements in ligating and dividing the cystic duct.

Significant changes in OPIs were not seen solely with ligation/division of the cystic duct. In the ligation/division of the cystic artery step, the number of times the master clutch was used increased in a statistically significant manner as more operations were performed. This result perhaps indicates that there was learning in how to use the finger clutch without inadvertently engaging the clips used in this step, or perhaps that later operations in the study were more complex and required larger movements and thus more positional adjustments. The latter may be true given that the only noticeable difference seen in the demographics was that in the third tertile, the acute cases, which were fairly evenly distributed amongst the tertiles, involved patients with the highest average BMIs, suggesting that later cases in this study may have been more difficult as the surgeon likely had to dissect through more adipose tissue in these operations.

There were also many significant findings for various kinematic OPIs, which include measurements of minute movements in the robotic arm (ex: angular velocity in each direction). A consistent finding seen in this study was significant changes in camera motion, particularly increasing speed, acceleration, and smoothness during the dissection of Calot's triangle, ligation/division of the cystic artery, and dissection of the gallbladder off the liver bed (Figure 2). It must be noted that a major difference between laparoscopic and robotic surgeries is that the surgeon is in control of the camera during robotic surgery. For a surgeon who is experienced in laparoscopy and then transitions to robotic surgery, learning how to control the camera is a major change in how the operation is conducted. Thus, these changes in camera speed, acceleration, and smoothness may represent a portion of the surgeon's learning curve for camera control. Interestingly, there were no significant changes found for camera motion in the ligation/division of the cystic duct step. We do not feel that there was a lack of learning; if positioned correctly, camera adjustments do not need to be made once the critical view has been established.

Surprisingly, there was no significant change in the duration taken to dissect Calot's triangle, as there is usually major attention paid to this step, and learning is thus expected to be a natural outcome. Importantly, the surgeon in this study was already well versed in laparoscopic cholecystectomy, and the techniques for laparoscopic and robotic dissection are very similar during this step. However, further analysis of the dissection of Calot's triangle reveals that there were consistent, significant changes seen in kinematic OPIs. Many increasing changes were seen in the angular speeds of the yaw and pitch joints of the wrist of the dissecting arm (Figure 3a) and in the angular speeds of the roll and pitch joints of the hand controller for the dissecting arm (Figure 3b). Although there were consistent increases in yaw and pitch of the dissecting arm, there did not appear to be significant changes in the linear movement of that arm. This finding of increased yaw and pitch velocity in a relatively motionless arm likely represents the surgeon learning to utilize the wristed motion of the robotic instrument instead of using it as a “straight stick” laparoscopic instrument. Thus, it appears that the complex, fine motions of the robotic instruments during the dissection of Calot's triangle are becoming better utilized in the early adoption of the robotic platform.

Because the learning curve for robotic cholecystectomy has not yet been established, and the CUSUM analysis has been used for laparoscopic cholecystectomy, we wanted to investigate whether a learning curve could be constructed for robotic cholecystectomy using this type of analysis. CUSUM analysis of individual surgical steps generated a traditional learning curve for the idle time of robotic arms during the ligation/division of the cystic duct (Figure 1b). During the idle time for this step, three clips are placed on the robotic clip applier, which is then exchanged for a pair of scissors. The robotic arm controlling the clip application needs to be taken out so the clips can be placed by technicians, and the arm is placed back in by the bedside assistant before the surgeon retakes control of the console and resumes the operation. This finding suggests that idle time, in addition to total active time of the robotic arms, is perhaps a useful proxy for how efficiently a step in cholecystectomy is performed, given that the decrease in the total time it took to ligate/divide the cystic duct approached significance (*p* = 0.057). Additionally, this learning curve suggests that proficiency in ligating/dividing the cystic duct is achieved when a surgeon adept in laparoscopic cholecystectomy performs their 22nd robotic cholecystectomy. This result is encouraging because it demonstrates that proficiency can be attained relatively quickly if surgeons have technicians, nurses, and assistants who can learn to efficiently perform instrument exchanges. This makes sense because the transition to robotic surgery was occurring for the entire operating room staff during data collection. Therefore, our finding of a learning curve in idle time may represent the learning proficiency of the entire surgical team.

However, conclusions from CUSUM curves are limited due to the natural bias in how they are used. The peak of the CUSUM curves often represents the point at which a surgeon reaches “proficiency” in a procedure, but this point is easily influenced by the total number of procedures that are evaluated (13). Therefore, an unbiased method to define the learning curve for robotic cholecystectomies is still being developed.

Given the growing use of robotic systems like the Da Vinci Xi to perform procedures like cholecystectomy, we felt it was essential to study how proficiency can be reached in robotic cholecystectomy. Currently, there is no way to track such proficiency, and OPIs have the promise to do so. Thus, understanding the proficiency of surgeons is of utmost importance, with the ultimate purpose of providing better goal-directed training to surgeons already experienced in the laparoscopic technique making the transition from laparoscopic to robotic cholecystectomy.

This study is limited in its implications. First, this study only included data from a single surgeon. As such, a multi-surgeon study may validate this study's findings. Next, this study did not include data from the first six robotic cholecystectomies that the surgeon performed. If that data had been accessible and thus included, perhaps the changes in OPIs would have been more pronounced because some learning was already done within the first six cases. Also, the sample size of 33 was limited, and future studies with larger sample sizes can have stronger implications. However, these data are valuable because the operations were not performed in conjunction with residents, minimizing confounders in the data collected.

Further studies are needed to better understand what factors contribute to greater proficiency in performing robotic cholecystectomies so that surgeons already experienced in the laparoscopic technique transitioning from laparoscopic to robotic cholecystectomies can be properly trained. We feel that the step of ligating and dividing the cystic duct during robotic cholecystectomy is a unique step that should be further evaluated for early learning curve changes for burgeoning robotic general surgeons.

## Conclusions

To our knowledge, this is the first study to investigate the learning curve for a surgeon experienced in laparoscopic cholecystectomy who is making the transition from laparoscopic to robotic technique. Our study identifies active time of robotic arms, use of the master clutch, and idle time as objective performance indicators (OPIs) that are worth tracking for proficiency. In addition, our study reveals significant changes in various kinematic measures associated with camera motion during multiple steps. Lastly, our study also reveals the ligation/division of the cystic duct, a step that involves a steep learning curve when transitioning from the laparoscopic to robotic platform, as a step worthy of further investigation for early learning curve changes in a surgeon transitioning from laparoscopic to robotic surgery.

## Data Availability

The raw data supporting the conclusions of this article will be made available by the authors, without undue reservation.
