# Supplementary material for: Early learning curve changes in objective performance indicators during robotic cholecystectomy
Source: Front Surg. 2025 Oct 10;12:1679666. doi: 10.3389/fsurg.2025.1679666 (PMC12550773; doi:10.3389/fsurg.2025.1679666)
Supplement: Supplementary file 1 [file Supplementaryfile1.docx]

| **Demographic** | **First Tertile (4 acute, 7 nonacute cases)** | **First Tertile (Acute Cases)** | **First Tertile (Nonacute Cases)** | **Second Tertile (4 acute, 7 nonacute cases)** | **Second Tertile (Acute Cases)** | **Second Tertile (Nonacute Cases)** | **Third Tertile (3 acute, 8 nonacute cases)** | **Third Tertile (Acute Cases)** | **Third Tertile Nonacute cases)** |
| --- | --- | --- | --- | --- | --- | --- | --- | --- | --- |
| Age (yrs) | 56.3±21.8 | 71.5±22.2 | 47.6±17.3 | 55.1±14.6 | 57.5±12.8 | 53.7±16.4 | 50.8±16.6 | 44.3±31.4 | 53.0±10.6 |
| BMI | 27.2±4.1 | 25.6±3.2 | 28.1±4.4 | 30.1±8.1 | 25.7±1.8 | 32.6±9.4 | 32.4±6.6 | 37.5±8.0 | 30.6±5.6 |
| Length of Surgery (min) | 72.0±36.5 | 106.3±42.4 | 52.4±9.8 | 55.7±22.6 | 68.0±33.8 | 48.7±10.9 | 59.4±18.0 | 80.3±20.6 | 52.4±11.0 |
| Time of recovery (hrs) | 1.5±0.8 | 2.2±0.9 | 1.1±0.4 | 1.9±0.8 | 2.0±0.6 | 1.9±0.9 | 1.6±0.4 | 1.8±0.5 | 1.5±0.3 |
| LOS in hospital (hrs) | 2.7±0.9 | 2.5±0.5 | 2.9±1.1 | 3.52±1.20 | 4.4±1.6 | 3.0±0.6 | 3.53±1.33 | 3.9±2.4 | 3.4±0.9 |
| ASA physical status | 2.36±0.5 | 2.25±0.5 | 2.43±0.53 | 2.45±0.69 | 2.50±0.58 | 2.43±0.79 | 2.50±0.67 | 3.0±0.0 | 2.33±0.71 |
| Post-op ED visits | 1 | 0 | 1 | 0 | 0 | 0 | 1 | 0 | 1 |

**Supplementary Table 1. Averages of Quantitative Demographics By Tertile.** The data is reported in the format “mean ± standard deviation”. This data represents quantitative demographics for all 33 cases and for acute and non-acute cases separately.


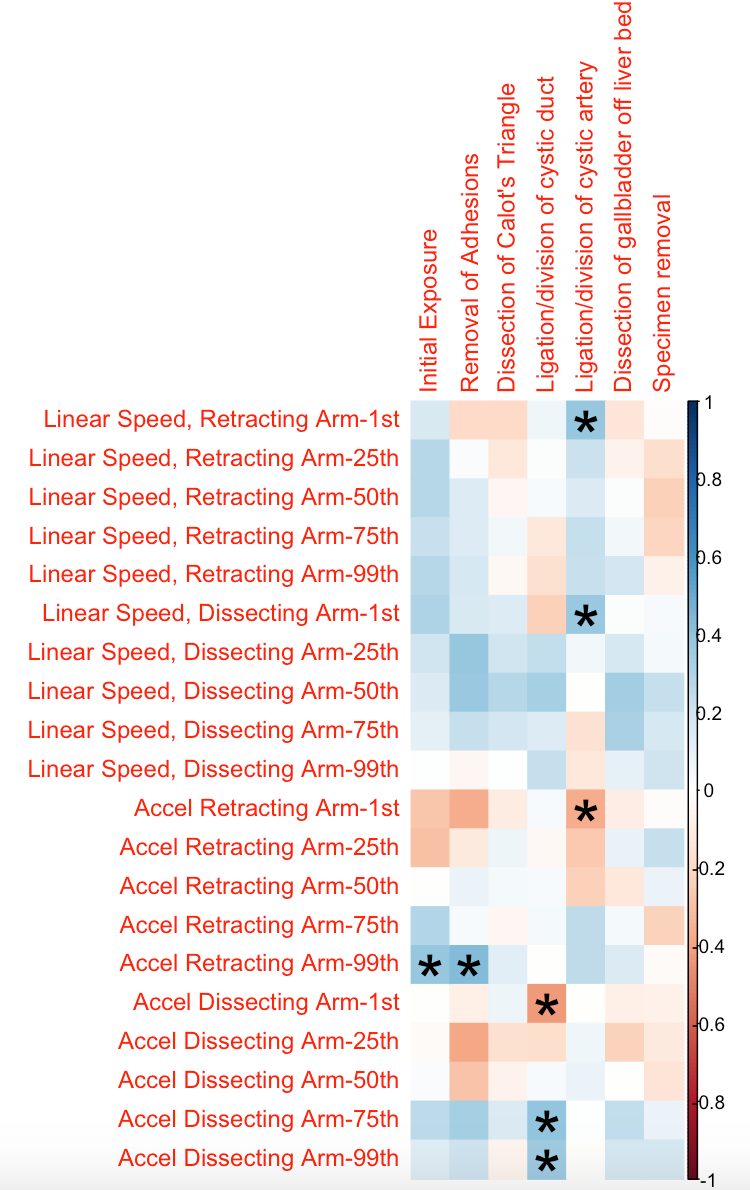


**Supplementary Figure 1. Correlation Plot Showing Strength of Correlation and Statistical Significance for Speed and Acceleration of The Retracting** **And Dissecting Arms.** The Kinematic OPIs are listed as row headings, whereas the steps of cholecystectomy are listed as column headings. A “*” in a box indicates statistical significance. “1st” represents the 1^st^ percentile for a given measure (same for “25th”, “50th”, “75th”, and “99th”). The key for strength of correlation is displayed towards the far right, with blue indicating positive correlations and red indicating negative correlations.


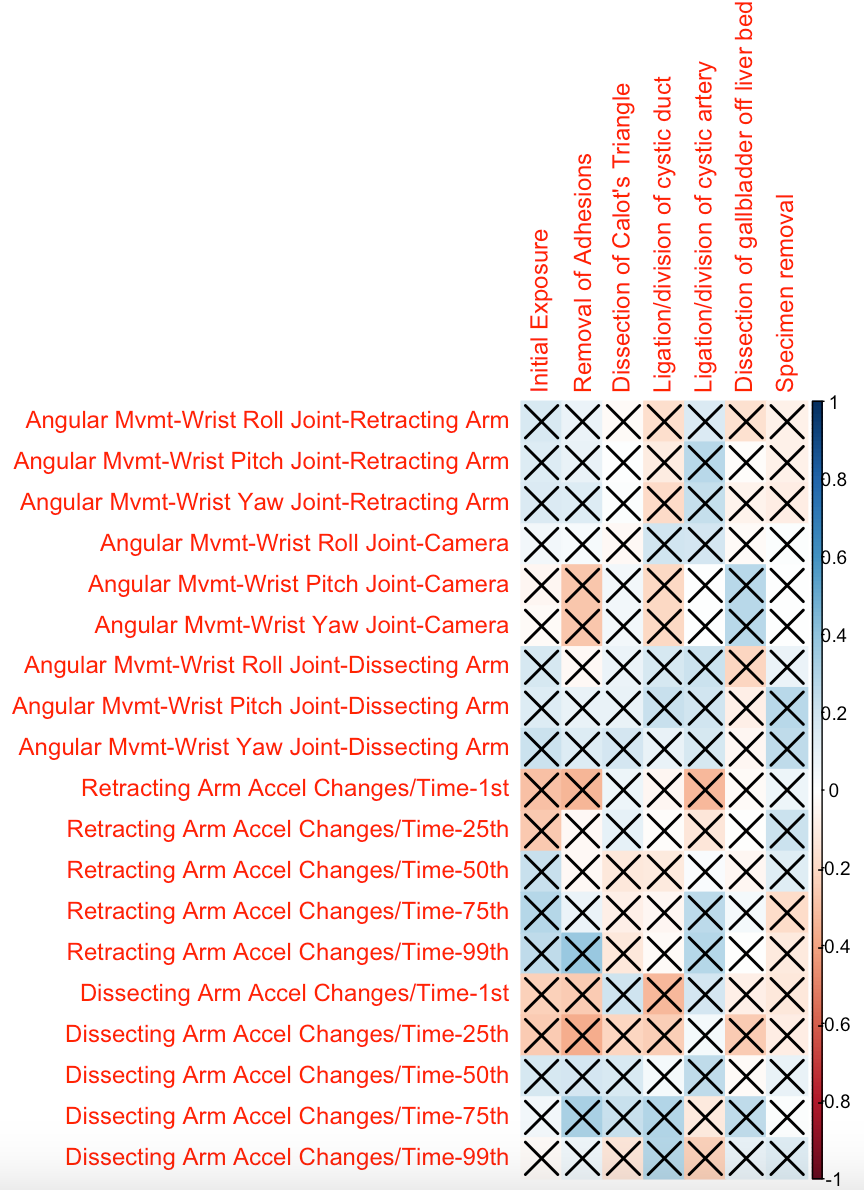


**Supplementary Figure 2. Correlation Plot Showing Lack of Significance of Various Kinematic OPIs.** The Kinematic OPIs are listed as row headings, whereas the steps of cholecystectomy are listed as column headings. An “X” in a box indicates lack of statistical significance. “1st” represents the 1^st^ percentile for a given measure (same for “25th”, “50th”, “75th”, and “99th”). The key for strength of correlation is displayed towards the far right, with blue indicating positive correlations and red indicating negative correlations.
